# Cross-talk between Rho and Rac GTPases drives deterministic exploration of cellular shape space and morphological heterogeneity

**DOI:** 10.1098/rsob.130132

**Published:** 2014-01-22

**Authors:** Heba Sailem, Vicky Bousgouni, Sam Cooper, Chris Bakal

**Affiliations:** Chester Beatty Laboratories, Division of Cancer Biology, Institute of Cancer Research, 237 Fulham Road, London SW3 6JB, UK

**Keywords:** cell morphogenesis, RNAi screening, image analysis, Bayesian learning

## Abstract

One goal of cell biology is to understand how cells adopt different shapes in response to varying environmental and cellular conditions. Achieving a comprehensive understanding of the relationship between cell shape and environment requires a systems-level understanding of the signalling networks that respond to external cues and regulate the cytoskeleton. Classical biochemical and genetic approaches have identified thousands of individual components that contribute to cell shape, but it remains difficult to predict how cell shape is generated by the activity of these components using bottom-up approaches because of the complex nature of their interactions in space and time. Here, we describe the regulation of cellular shape by signalling systems using a top-down approach. We first exploit the shape diversity generated by systematic RNAi screening and comprehensively define the shape space a migratory cell explores. We suggest a simple Boolean model involving the activation of Rac and Rho GTPases in two compartments to explain the basis for all cell shapes in the dataset. Critically, we also generate a probabilistic graphical model to show how cells explore this space in a deterministic, rather than a stochastic, fashion. We validate the predictions made by our model using live-cell imaging. Our work explains how cross-talk between Rho and Rac can generate different cell shapes, and thus morphological heterogeneity, in genetically identical populations.

## 2. Introduction

Cell shape results from dynamic interactions between the cytoskeleton, cell membrane and adhesion complexes that interface with the extracellular environment, often via the actions of regulatory signal transduction systems [1–4]. Form follows function, and specific shapes are essential for particular cellular behaviours such as migration. For example, in many cells, motility is generated by formation of filopodial and lamellipodial protrusions at the leading edge (LE), which become sites of extensive adhesion to the underlying substrate generating traction, while at the trailing edge (TE), high contractility and the disassembly of adhesions generate a propulsive force [5]. Thus, efficient migration requires that a cell ‘finds’ the appropriate set of migratory shapes from a space of all the shapes it could possibly assume [6]. Deterministic or stochastic searches of shape space are also likely to underpin the morphological heterogeneity of different genetically identical populations [7]. But very few quantitative or qualitative descriptions exist of any cell's shape space, how a cell explores this space and genes that regulate these searches.

In all eukaryotic cells, Rho-family GTPases dynamically control cytoskeletal organization to regulate cell shape [8]. Rho-family GTPases promote localized changes in cell morphology, as well as coordinating shape changes over the entire cell [9,10]. In cells migrating using cycles of protrusion, adhesion and retraction, activation of RhoA promotes both adhesion disassembly at the TE as well as cell-wide tension that is critical to restricting protrusion at the LE [10–13]. Moreover, in some migrating cells, RhoA is activated at the LE synchronous with cell advancement and is spatially segregated from a proximally localized wave of Rac1 and Cdc42 activity [14]. The fact that compartmentalized regulation of Rho-family GTPase activity is critical for generating particular shapes is highlighted by experiments which show that migration is inhibited when Rac activation is uniformly distributed throughout the cell [11,15–17]. Local Rho-family GTPase activity is established by the actions of Rho GTP exchange factors (RhoGEFs) and Rho GTPase activating proteins (RhoGAPs), which are recruited by upstream signals to distinct subcellular milieus [18,19]. There is considerable cross-talk between Rac- and Rho-type GTPases. For example, at the LE Rac1 inhibits Rho1 by activating p190RhoGAP [20–22]. Conversely, Rac1 can also upregulate RhoA activity [23,24], which may reinforce the ability of LE protrusions to suppress the formation of protrusions at the TE via upregulation of tension [11,25].

While classical genetic and biochemical studies have begun to describe mechanisms central to morphogenesis at detailed molecular levels [3], given the complexity of the processes involved it is not possible to predict how the activity of these components generates particular shapes using solely bottom-up approaches [13]. Here, we describe the implementation of top-down approaches to gain insight into the exploration of shape space by migrating cells. We first determine the shape space explored by *Drosophila* BG-2 neuronal cells using a dataset where the morphology of single cells has been quantified following systematic RNAi (RNA interference) and/or gene overexpression [26]. We find that wild-type BG-2 cells adopt six discrete shapes, and only rarely adopt a seventh shape even following gene depletion. Next, we generate two complementary models: a Boolean model explaining the biochemical basis for different cell shapes, and a Bayesian model predicting the next shape a cell will adopt based on its current shape. These models demonstrate that the cross-talk between Rac and Rho drives the deterministic exploration of shape space, and underpins the morphological heterogeneity of cellular populations.

## 3. Results and discussion

### 3.1. A state space defined by seven different shapes

To quantify the number of cell shapes that can be adopted by a motile metazoan cell, we made use of a dataset where we previously quantified the cell shape of both wild-type *Drosophila* BG-2 cells, and BG-2 cells after systematic RNAi and/or gene overexpression of different cytoskeletal components and regulators, including Rho-family GTPases, RhoGEFs and RhoGAPs [26]—termed treatment conditions (TCs) [26]. For the analysis described here, we have data for 256 different TCs; this includes seven more TCs than our original analysis. *Drosophila* BG-2 cells are a neuronal migratory cell line that form integrin–extracellular matrix (ECM)-based adhesions and cell–cell adhesions [27,28]. Migratory BG-2 cells generate extensive filopodial [29] and lammellipodial protrusions, and the LE assumes a ‘fan-like’ shape (see electronic supplementary material, movie S1). While the TE of motile BG-2 cells contracts during migration, these cells exhibit a long ‘tail’ at the TE (see electronic supplementary material, movies S1 and S2). In culture, BG-2 cells migrate in a processive manner in one direction for relatively short (more than 1 h) periods of time before altering their direction (see electronic supplementary material, figure S1).

In our previous analysis of this dataset, we generated 145 features that describe the geometry, protrusion and the distribution of GFP intensity of each cell using a MATLAB toolbox that was developed in-house (CellSegmenter) [26]. We then used a supervised method that first classifies single cells according to their similarity to different ‘reference’ or ‘exemplar’ phenotypes to generate a quantitative morphological signature (QMS) for each cell. Finally, we clustered the average QMS of cell populations (e.g. following depletion of a particular gene by RNAi) to group different TCs, and thus describe gene groups, or ‘local networks’, that contribute to the regulation of different morphological processes [26]. However, there are a number of aspects to this type of analysis that make it unsuitable for determining cellular shape space and for generating predictive models: (i) reference shapes were chosen manually as phenotypic extremes [26], and therefore it is possible that the shape space defined by these phenotypes does not account for the variance present in the dataset; (ii) as the reference shapes are generated by overexpression of constitutively active forms of different Rho-family GTPase or RhoGEFs, the space defined by the shapes may in fact not represent a physiologically meaningful one [26]; (iii) owing to the fact that QMSs are normalized to the control TC—enhanced green fluorescent protein (EGFP) alone—this initial analysis provides no insight into the actual space explored by wild-type cells; and (iv) all clustering was performed using the average QMSs of different populations, which does not account for the morphological heterogeneity of populations, and each average QMS or cluster of QMSs may not represent the shape space explored by individual cells [7,30,31].

**Figure 1.**
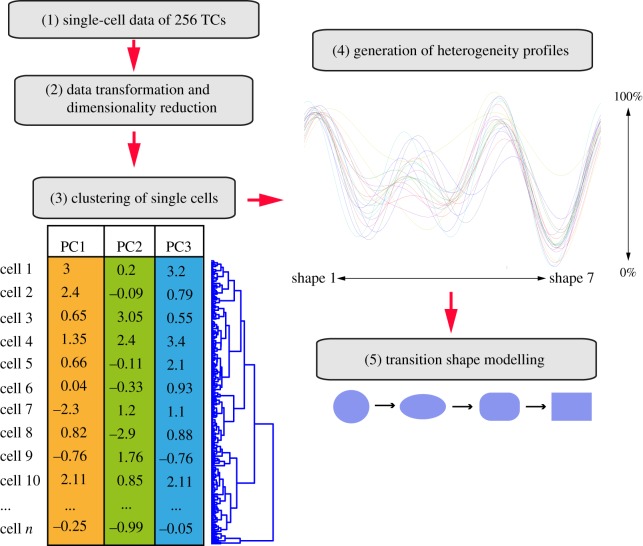
Workflow for quantifying cellular shape space. (1) A high-dimensional dataset that measures 145 morphological features of 256 TCs and 12 061 cells is (2) log-transformed and projected into the first three principal components (PCs). (3) Clustering of single cells is performed and results in seven distinct shapes. (4) For each TC, the frequency of each shape in the population is calculated and normalized to wild-type cells (cells expressing EGFP alone), resulting in a normalized TCHP. The distribution of 20 TCs in the seven shapes is shown. (5) A transition model is built using Bayesian learning to learn the order between shapes.

**Figure 2.**
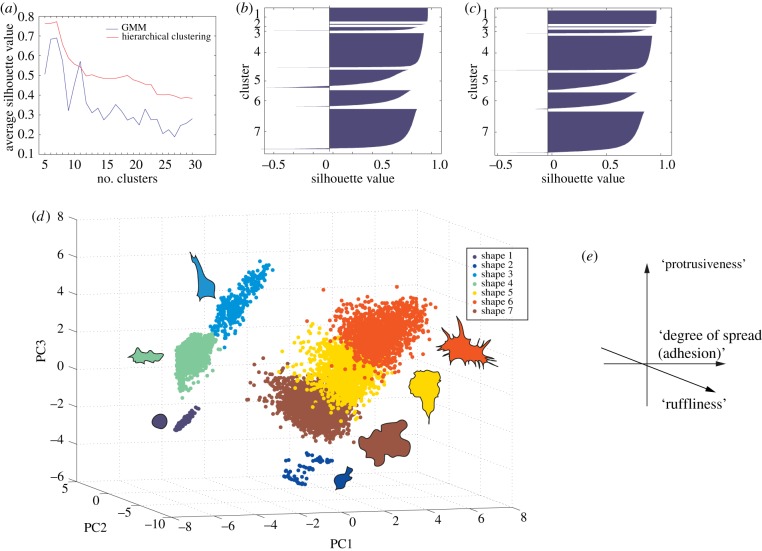
Single-cell clustering. (*a*) Average silhouette value for different numbers of clusters using Gaussian mixture modelling (GMM) and hierarchical clustering. Higher averages represent better cluster quality, and the best clustering for this dataset was reached when cells were grouped into seven clusters using hierarchical clustering. (*b*) Silhouette values of single cells for the best model. (*c*) Silhouette values of single cells for the best model after correction using KNN. (*d*) Single-cell data for all TCs are projected in the first three PCs and coloured based on the single-cell hierarchical clustering results, where clusters are denoted by shapes 1–7. Next to each shape cluster is a representative cell shape from that cluster. (*e*) Qualitative interpretation of PC space.

In order to determine the number of cell shapes present in both wild-type cells, and following systematic RNAi, we implement unsupervised classification methods that consider the heterogeneity of single cell, and not averaged, populations (figure 1). We first scale and log-transform the data where single cells are each described by 145 normalized features. Because many of these features are correlated and/or noisy, we use principal component analysis (PCA) as a data reduction method (Material and methods). To identify the number of distinct phenotypes in the data, we divide all cells in the dataset into 5–30 groups, or 25 cluster models, using two clustering methods: hierarchical clustering and Gaussian mixture modelling (GMM). We then assess the quality of clustering in the resulting 50 cluster models using the average silhouette value of each model. The silhouette value measures how similar a given cell is to other cells in the same cluster, and to cells in other clusters. A model with high average silhouette value indicates that cells in each cluster are similar to each other and dissimilar from cells in other clusters. Thus, a higher silhouette value reflects the robustness of the clustering at different thresholds. Initial hierarchical clustering results in higher average silhouette values than GMM at all thresholds, and the best performance is achieved when cells are grouped into seven clusters by hierarchical clustering (figure 2*a*). Nevertheless, a plot of silhouette values for hierarchical clustering shows that some cells are misclassified, as indicated by their negative silhouette value (figure 2*b*). In this case, misclassification may be due to the fact that hierarchical clustering assigns points to clusters in one pass. Therefore, cells that are incorrectly assigned to a cluster in the first pass will not be re-assigned. We thus reclassify cells with a low silhouette value (less than 0.6) using the *k*-nearest neighbours (KNN) algorithm (Material and methods), which increases the average silhouette value (figure 2*c*). This analysis reveals that the morphological space that is explored by BG-2 cells across 256 different TCs is indeed best defined by seven different shapes (figure 2*d*; electronic supplementary material, figure S2). Through qualitative assessment of the different shapes in principal component (PC) space and examination of the features which make large contribution to each PC, we propose PC1 captures the extent of spread (or adhesiveness), PC2 captures cell ‘ruffliness’, whereas PC3 captures the extent of protrusiveness (figure 2*e*; electronic supplementary material, tables S1 and S2). The high average silhouette values of the corrected hierarchical clustering model suggests that these seven shapes are potentially discrete forms and not continuous variations of each other.

**Figure 3.**
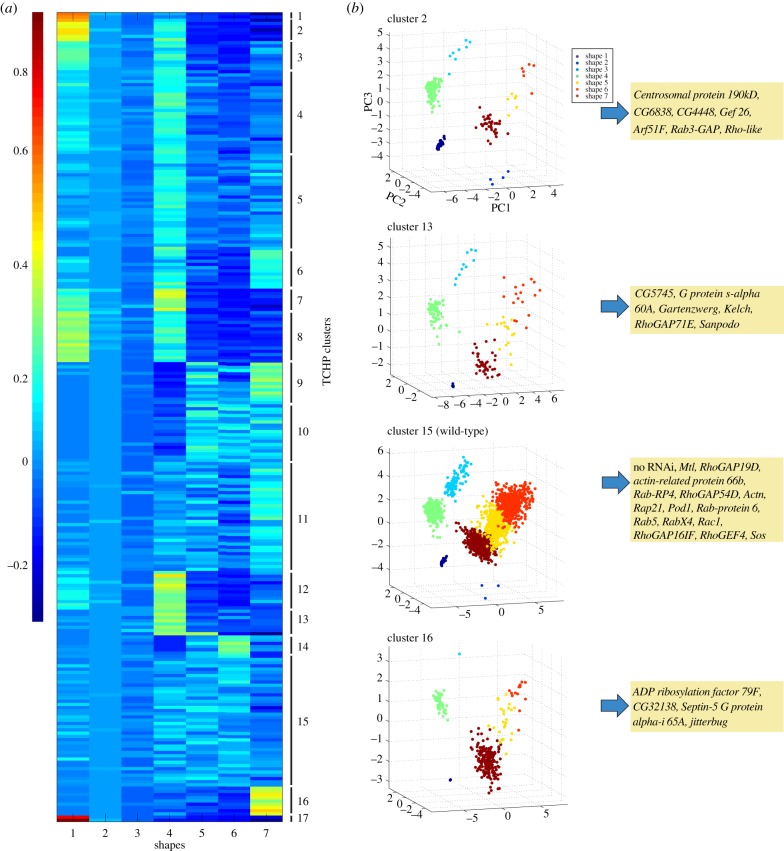
Clustering of normalized TCHPs. (*a*) Heatmap of normalized TCHPs and their clustering based on the increase/decrease in different shapes. Red colour indicates high frequency of a shape in a TC, dark blue colour indicates low frequency of a shape in a TC. We identified 17 clusters in total. (*b*) Single cells from different clusters were plotted in three-dimensional shape space. Each colour represents a different shape. Representative TCs from different clusters (all RNAi) are listed in yellow boxes.

We quantify how gene depletion or overexpression affects the ability of BG-2 cells to adopt these seven shapes using treatment condition heterogeneity profiles (TCHPs). A TCHP is a seven-dimensional vector describing the proportion of each of the seven shapes in a TC. Interestingly, wild-type BG-2 cells are notably heterogeneous, as six of the seven shapes that exist in the entire dataset are present in control TC (3% shape 1, 0% shape 2, 6.9% shape 3, 20% shape 4, 17% shape 5, 18.6% shape 6 and 33.8% shape 7). In order to account for the basal heterogeneity of wild-type BG-2 cells, and reflect the difference between the normal and perturbed populations, we normalize all TCHPs by subtracting the wild-type profile. We then cluster normalized TCHPs to identify TCs with similar effects on the distribution of different cellular shapes. Clustering using classical distance measures (such as Euclidean or City Block) gives equal weight to the differences in each shape. However, some shapes are closer to each other in PC space (e.g. shapes 4, 5 and 7) than others (e.g. shapes 1 and 2). To give weight to such differences, we use a distance metric method that incorporates the difference between the cellular distribution and the difference between the cluster/shape means (Material and methods). The use of this method generates 17 TCHP clusters (figure 3*a*; electronic supplementary material, table S3). Cluster 15 contains wild-type cells, and all TCs that can be considered controls, as they are similarly heterogeneous (i.e. these populations have the same six shapes in the same relative proportions as wild-type cells). Interestingly, the morphological heterogeneity of populations is decreased in the vast majority of TCs, most of which are composed of one to four shapes that are also present in the wild-type population (figure 3*a*; electronic supplementary material, table S3). Thus in these more homogeneous populations, specific shapes that exist in the wild-type populations have become highly enriched, and new mutant shapes do not typically emerge (a shape is considered enriched in the population if it comprises more than 10% of the population). Only in a handful of cases did cells adopt a seventh shape (shape 2) in addition to adopting a subset of the six wild-type shapes, although shape 2 is not considered enriched in any TC (see electronic supplementary material, table S3). We propose that decreased heterogeneity following gene inhibition or overexpression is because cells become ‘trapped’ in particular shapes during normal exploration of shape space. Our finding that BG-2 morphogenesis occurs in a low-dimensional space where heterogeneity is most often decreased by genetic perturbation is consistent with our recent finding that *Drosophila* Kc haemocyte cells and metastatic melanoma cells adopt a limited number of discrete shapes even following RNAi [7], as well as with the work of Keren *et al*. [13], who show that migrating fish keratocytes adopt a limited number of shapes. The methods we use to describe heterogeneity are similar to those implemented by Slack *et al.* [32] to examine the heterogeneity of different signalling events in isogeneic populations; however, to our knowledge, this is the first study to implement heterogeneity profiles to describe cell shape in the context of an RNAi screen.

### 3.2. Assessment of different cell shapes

In order to understand the mechanistic basis for the different cell shapes adopted by BG-2 cells, we first qualitatively characterize each of the seven shapes present in the entire dataset based on three broad parameters: polarity, protrusivity, and the extent to which cells are spread and appear adhered to the underlying substrate (table 1). Furthermore, when possible, we infer the activation state of either Rac-type GTPases (hereafter referred to as ‘Rac’; we cannot differentiate here between the activity of Rac1, Rac2 and Mtl) or Rho1 GTPases (the *Drosophila* orthologue of mammalian RhoA), based on those genes whose depletion or overexpression results in a particular shape, and in some cases the protrusive/contractile nature of the cell. For example, shape 5 is a polarized shape characteristic of motile cells, where cells exhibit lamellipodia and/or filopodia at the presumptive LE, and a contractile tail at the TE. Based on previous literature [9], we assume that in shape 5 cells Rac activity is high at the LE, and Rho activity is high at the TE. In support of this idea, shape 5 is decreased following Rho or Rac activation or inhibition (see electronic supplementary material, table S3). By contrast, shape 1 is enriched following Rho activation, and shape 7 is enriched following Rac activation. Thus, different states of Rho/Rac activity correlate with different shapes and with the activation of specific morphological processes such as protrusion or adhesion.

**Table 1.** Characterization of each shape and TCs enriched in each shape.

shape	polar?	protrusive (filopodia and/or lamellipodia)	spread? (adhesive state)	characteristic genes	Rac	Rho
1	no	no	no, very round	DNRhoGEF3 overex, G65A overex, *CG33232*	low	very high
2	mildly	very small, ‘bleb-like’ protrusions	no, rounded	*cpa*, *kat-60L1*, *RecQ5*		high^a^
3	mildly	yes, biopolar, thin protrusions	long cells	*RhoGAP71E*, *Rab3*, *CG5522*, *Rab3-GAP*		
4	yes	poorly formed lamellipodia	poorly spread	*cappuccino*, *Sop2*, *RagC*, *GXIVsPLA2*, *Nf1*, *trio*	low	
5	yes	LE filopodia and lamellipodia	presumptive LE well attached, while presumptive TE is contracted	*sandopo*, *RhoGAP100F*, *CG10724*, *CG33275*, *RhoGAP102A*, wild-type	high (LE)	high (TE)
6	no	yes, filopodia and lamellipodia	yes, multiple sites of adhesion	*RhoGEF2, RhoGAP93B*, *Cdc42*, *memo*, *CG8557*, *RhoGAP92B*	high	low
7	no	yes, flat lamellipodia	yes, large well-spread	*Sept5*, RacV12 overex, RacF28L overex	very high	low

^a^Inferred on the basis that shape 2 is very similar to shape 1.

### 3.3. A Boolean model to describe cell shape by localized Rho/Rac activation

**Figure 4.**
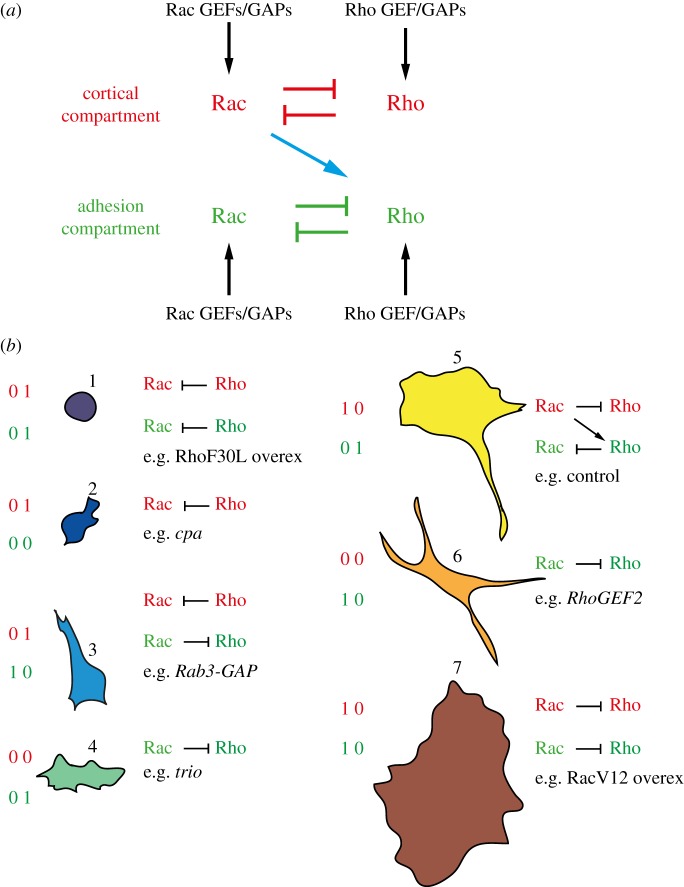
A simple model of Rho/Rac activity in two distinct compartments exists in seven states. (*a*) We consider Rac and Rho activity in the cortical (red) and adhesion (green) compartments. In both compartments, Rac and Rho antagonize each other. Cortical Rac activity can also activate Rho in the adhesion compartment (blue arrow). In both cortical and adhesion compartments, both Rac and Rho can be inactive. Alternatively, the state of the network can be determined by upstream signals such as RhoGEFs and RhoGAPs, and thus the model is non-autonomous. (*b*) By comparing how gene depletion or overexpression leads to the enrichment of specific shapes (figure 3 and table 1), we match the seven possible shapes to the seven possible network states of the model.

Given that each of the seven shapes correlates qualitatively with different levels of Rho/Rac activity, and/or differences in protrusiveness/ruffliness and cell spreading (table 1 and figure 2*e*), we reason that morphological variance in the entire dataset may be explained by the action of Rac and Rho GTPase acting locally to regulate cytoskeleton organization. We thus devise a simple Boolean model [33] to explain how spatial differences in Rho/Rac activity lead to specific shapes based on known literature and various TC profiles. In this model, we separate signalling activity into two non-identical compartments: a compartment regulating cortical morphology and another regulating adhesion (figure 4*a*). We consider the cortical compartment to broadly include cellular regions where cytoskeleton rearrangements that drive protrusions (e.g. blebbing, filopodia, lamellipodia) or retraction occur. Rho/Rac activity can be considered ‘on’ (1) or ‘off’ (0) in each compartment (figure 4*a*). In the cortical compartment, Rac activity drives protrusion, whereas Rho promotes contractility. In the adhesion compartment, Rac promotes the formation of focal adhesions, whereas Rho upregulates tension and ultimately adhesion disassembly. We also make the assumption that in each compartment one activity antagonizes the actions of the other (figure 4*a*) [22,34,35], and that Rac or Rho activity require upstream activation in either compartment. Thus, in the absence of such activation both Rac and Rho can be off, although either Rac or Rho must be active at any time in either compartment. Finally, our model assumes that Rac-mediated protrusion in the cortical compartment promotes Rho-mediated retraction in the adhesion compartment (figure 4*a*), incorporating observations that polarized protrusivity can promote retraction in the opposing end via upregulation of tension, and that Rac can activate Rho [11,25]. However, the reverse process does not occur. Although Rac activation in the cortical compartment can activate Rho in the adhesion compartment, local inactivation of Rho by active Rac in the adhesion compartment predominates over this global effect. Notably, because we allow upstream regulators (such as RhoGEFs and RhoGAPs) to influence the activation state of our model at any given time, this model does not engage in autonomous state cycles, and thus cannot be used by itself to predict transitions between shapes. While the predictive power of our model was not quantitatively compared with alternative Boolean models with different wirings, it is entirely consistent with existing literature and requires few assumptions.

Our model of Rho/Rac-mediated control of cell morphology can exist in only seven different possible states of activation at any given time (figure 4*b*), a number that corresponds exactly with the number of cell shape clusters with the highest silhouette index value (figure 2*d*). We are able to match each possible state of the model with different shapes based on the degree of each shape's protrusivity/contractility and adhesiveness, as well the genetic background that enriches for a particular shape (figure 4*b*). For example, based on extensive literature we infer that Rac activation predominates in the cortical compartment of shape 5 cells (i.e. at the LE), and Rho activity is high in the adhesion compartment, especially at mature focal adhesions [10,36]. Furthermore, cells overexpressing constitutively active versions of Rac (shape 7) or Rho (shape 1) can be assigned to states where GTPase activity is high in both cortical and adhesion compartments (figure 4*b*). Shape 2 is very related to shape 1, and we assume there is little Rac activity in these mostly rounded cells (figure 4*b*). Depletion of the Rho1-specific GEF *RhoGEF2* [37] or dysregulation of polarity following depletion of *Cdc42* [38] enriches populations in shape 6, suggesting that this shape is due to defects in Rho activation in the cortical compartment (e.g. due to failure of Rho1 to be activated by RhoGEF2) coupled with inappropriate Rac activation in the adhesion compartment. Shape 4 cells exhibit defects in lamellipodia formation following depletion of genes such as the Rac GEF *Trio* [39], presumably owing to the inhibition of Rac-mediated protrusivity (figure 4*b*). Shape 3 cells are rare, and it appears that on the one hand these cells are contractile (because of Rho activity), yet do not round-up completely like shape 1 cells because Rac is promoting adhesion formation. That we can match different shapes to different activation states of the model supports the idea that the seven shapes in our dataset are driven predominantly by balancing Rac and Rho activity in two distinct compartments.

### 3.4. Morphological heterogeneity as a structured process

When cells are in a given shape (e.g. shape 1), do they randomly assume any other shape? Or is the adoption of one shape dependent on the prior adoption of another shape, such that there exists particular deterministic ‘routes’ in shape space to which cells are constrained? While our Boolean model explains the basis for the different shapes it cannot be used to understand how BG-2 cells explore shape space, because once a cell is in a certain state in this model it is allowed to switch to any other state, as Rac and Rho in different compartments are activated or inhibited by upstream factors such as RhoGEFs and RhoGAPs, whose activity may change in ways that are not predicted by the model. Thus, in order to determine whether BG-2 cells explore shape space in a stochastic or deterministic fashion, we use Bayesian learning to generate an acyclic graphical representation, or network, of probabilistic dependencies between shapes [40]. In this network, each shape is a node, and the dependency of a downstream node on an upstream node is represented as an arc from the parental node to the downstream child node. These dependencies can be interpreted as causal influences of the parent on the child. An attractive property of this method is that it enables the representation of the nonlinear relationships between variables. This tool has proved to be successful in modelling signal transduction cascades [40–43], but to date has not been used to model cell morphogenesis.

**Figure 5.**
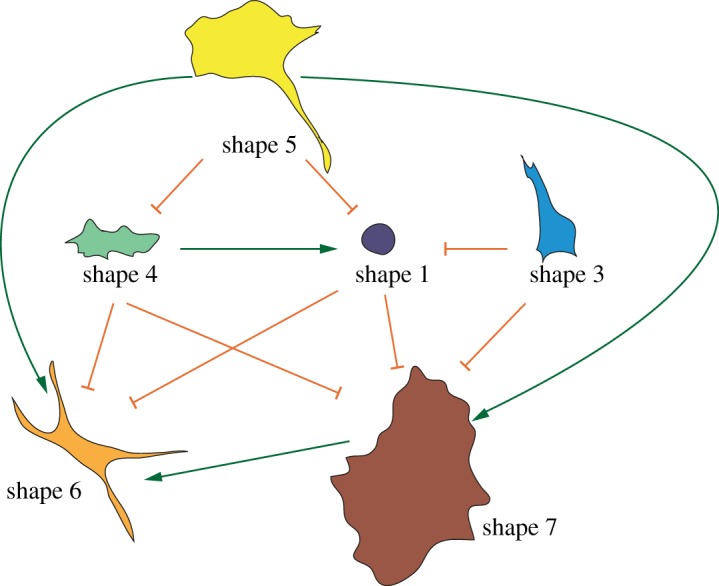
Exploration of shape space by BG-2 cells occurs in a deterministic manner. Shape transition model: arrows describe the observed dependency of one shape on another. Green arrows describe dependencies where the correlation between shapes is positive; orange arrows describe dependencies where the correlation between shapes is negative.

In our case, each TC has a certain proportion of cells in each shape. These shapes can be treated as variables, and their dependency, if any, can be inferred using the interventional cues (here RNAi-mediated gene depletion) that enable the identification of the directionality between different shapes. For example, if cells must pass through a shape X before becoming a shape Y, then any TCs where there is a decrease in shape X will also result in a decrease in shape Y. Conversely, perturbations that inhibit only the transition to shape Y will not affect shape X, which would be reflected in the TCHP as a decrease in shape Y but not shape X. In fact, shape X may accumulate if transitions to shape Y are blocked. This method does not require us to pose any assumptions about the distribution of these variables. Here, the most probable model is quantitatively determined from a number of other alternative models, and if no dependencies exist a network will not be generated. Using the 256 TCHPs, we generate a transition model between different shapes (figure 5). Notably, not all shapes are necessarily incorporated into the model. For example, shape 2 is not part of this model as its presence does not positively or negatively correlate with any other shape. That we can generate a dependency model of six shapes strongly suggests BG-2 cells explore shape space in a deterministic fashion, and that morphological heterogeneity of BG-2 cells is a structured process.

At the top of the hierarchy are polarized shape 5 cells (figure 5). The spread morphologies of shapes 6 and 7 are dependent on the migratory shape 5, suggesting that migration is inhibited by reactivation of Rac at adhesions, particularly at the TE, resulting in a loss of a polarity. One caveat to the use of Bayesian learning methods is that they cannot infer cyclical relationships, so it is possible that shape 5 is also dependent on shapes 6 and 7, although this is less probable than the observed dependencies.

We further examine the nature of the dependencies by examining the correlations between different shapes. Positive correlations indicate that correlated shapes are often simultaneously present in the same TC, while negative correlations indicate that the presence of one shape predicts the depletion of another. For example, the presence of shape 5 in a population predicts shapes 1 and 4 are less likely to be present. Moreover, the presence of shapes 1, 3 and 4 predicts that shapes 6 and 7 will not be found in the population. Thus, when cells are driven to adopt shapes 1, 3 and 4, either by a cue or following genetic perturbation (e.g. RNAi), they are unlikely to adopt another shape. These findings suggest that while morphogenesis is a deterministic dynamic process, particular regions of shape space can trap cells in a given shape.

**Figure 6.**
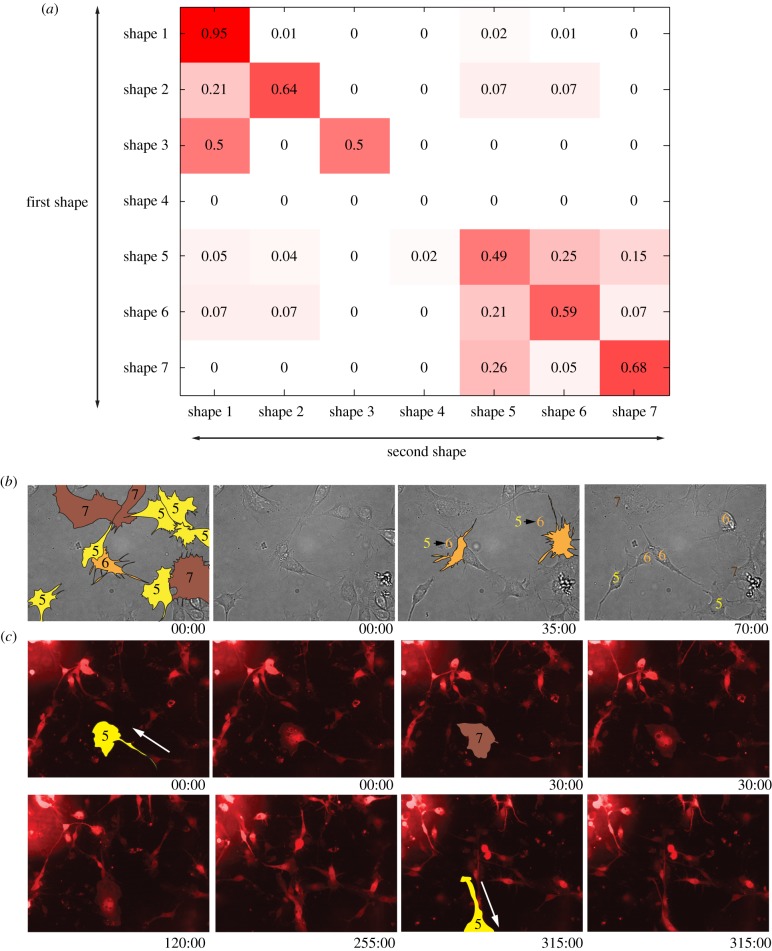
Validation of the Bayesian model by live-cell imaging. (*a*) The probability of a cell transitioning from an initial shape (rows) to a subsequent shape (columns). The diagonal describes situations where cells do not transition over the course of the imaging experiments (*n* = 200 transitions). (*b*) BG-2 cells were imaged by brightfield microscopy for 70 min. At 35 min, two cells undergo transitions from shape 5 to shape 6. In total, the shape 5–6 transition occurs with a probability of 0.25. (*c*) BG-2 cells transiently expressing EGFP (shown in red) grown in standard culture conditions and imaged for 315 min. At the beginning of the experiment, the yellow-shaded cell is in shape 5, and at 30.00 min becomes shape 7; at 315 min the cell repolarizes in another direction and migrates as shape 5.

Quantifying transitions between shapes in living cells (Material and methods) reveals that while transitions between shapes are rare, they occur with frequencies consistent with the predictions made by our Bayesian model. Over a period of approximately 3 h, we observe that cells in any shape are most likely to remain in that shape (figure 6*a*). In fact, consistent with the model, shape 1 is the most stable shape, and rarely makes any transitions (figure 6*a*; electronic supplementary material, figure S3). By contrast, transitions between shape 5 and shapes 6/7 (figure 6*b*) are relatively frequent as the probability of transitions from shape 5 to shape 6 is 25%, and the probability of transition from shape 6 to shape 7 is 15%; and both observations are in accord with our model. Finally, as predicted, transitions between shapes 1–3 and shapes 5–7, or vice versa, rarely occur. Taken together, quantification of shape transitions in live cells validates our Bayesian model.

Notably, we do observe that the probability of shapes 6/7 transitioning to shape 5 is relatively high (figure 6*a*), which is not captured by our model, probably owing to the acyclic nature of Bayesian networks. In particular, we note transitions from shape 5 to shapes 6/7 occur when cells make large changes in the direction of their migration (figure 6*c*).

## 4. Conclusion

Through quantitative analysis of single-cell morphology following systematic RNAi, we show that wild-type *Drosophila* BG-2 cells adopt six distinct shapes in culture. We also show that most genetic perturbations only modify the distribution of the six wild-type shapes rather than generating new shapes [7]. In fact, the morphological variance present in the entire dataset is best described using only seven shapes. That disruption of biochemical networks regulating cellular morphogenesis typically decreases morphological heterogeneity in *Drosophila* cells, as demonstrated here and in our previous work [7], suggests that genes have evolved to promote and regulate morphological heterogeneity. By maintaining several different shapes simultaneously within the same isogenic population this may facilitate different single-cell and population-level behaviours. For example, specific subpopulations may be better suited for unexpected changes in environmental conditions (e.g. one population may be better suited for migration in a particular environment). Alternatively, different shapes may have different functions/behaviours, which promote survival of the population as a whole.

We provide a Boolean model that explains how Rho and Rac signalling in two distinct cellular compartments can drive the adoption of exactly seven shapes, and the morphological heterogeneity of different populations. A complementary Bayesian model shows that BG-2 cells make deterministic, and not stochastic, transitions between cell shapes. We propose that genetic perturbations affect deterministic transitions between shapes. For example, TCs where Rac signalling is suppressed result in the adoption of a round shape, and inhibit the transition to other protrusive shapes (shapes 5–7). The downside of using Bayesian networks in this context is that they cannot identify loops between shapes, and thus such models should only be taken as a suggestion of the system behaviour. Nonetheless, we are able to confirm a number of the predictions made by our model using live-cell imaging (figure 6*a*).

A prediction made by Bayesian inference (figure 5), and supported by live-cell imaging (figure 6*a*), is that there exist two primary attractor regions in the shape space explored by BG-2 cells. When conditions drive cells to a region of shape space where cortical tension is high, adhesion is low and cells are round, cells largely remain in this space. By contrast, when adhesion increases and cortical tension decreases, and cells become more spread, cells largely remain in this region of shape space (figure 6*a*). This finding is consistent with the fact that in culture, BG-2 cells have a propensity to either grow in the same culture as spread cells in a monolayer that make attachments to the ECM, or as ‘colonies’ of rounded cells that make poor cell–ECM contacts, but extensive cell–cell contacts (see the electronic supplementary material, movie S3 for an example). Potentially, these two cell shapes represent two diverse cell states (such as a stem-like and differentiated population) that have different migratory, metabolic and proliferative behaviours. Thus, transition between the two shapes, and thus attractor states, could require extensive transcriptional and/or epigenetic reprogramming. Regardless of what the different nature of these shapes may be, it is clear that Rho and Rac activity plays a role in dictating the transition between these shapes, and the morphological heterogeneity of the population.

We caution that our models, while providing insights into the exploration of shape space by Rho and Rac, are only applicable to a certain set of network states that are dictated by the genetic background and culture conditions. For example, our Bayesian model suggests that it is more probable for a migrating BG-2 cell to lose polarity than establish polarity, probably reflecting the fact that these cells are transiently polarizing and migrating for short periods of time in response to external signals that are not emanating from one direction in a consistent manner. However, if cells were exposed to a signal that generated directional migration, it would become more likely that these cells would transition from exploratory shapes to polarized shapes. Moreover, we stress that our model reflects cellular transitions with higher probabilities, and that transitions not described in the model can occur, but probably at lower frequencies. For example, by live-cell imaging we observe a number of transitions from shapes 6 and 7 to shape 5 (figure 6*a,c*). Our model predicts that once cells become rounded, it is unlikely they will become spread and/or protrusive, and the reverse is also true. But clearly transitions from rounded to protrusive shapes must occur—notably during the rare transition from a rounded mitotic shape to a spread shape following cytokinesis. Importantly, the analytical pipeline we present here can be used to generate models of cellular morphogenesis using diverse data sources, and thus it would be interesting to regenerate these models using images acquired of BG-2 cells cultured in different conditions, and/or different cell types.

Our Boolean model of shape does not require Cdc42 activation to explain cell morphology. Based on a number of previous studies, we propose that Cdc42 activation has an important role in establishing cellular polarity relative to external cues [44,45], which then results in differential Rho/Rac activity, but has little influence on controlling protrusion or adhesion. Thus, we hypothesize Cdc42 dictates the directionality of a migrating cell, but does not influence the overall shape. However, we cannot formally exclude the possibility that Cdc42 activity and other GTPases make significant contributions to morphogenesis beyond the establishment of polarity. But we note that BG-2 cells are different from many cells types in that filopodia formation is largely driven by a Rac-SCAR versus Cdc42-WASP pathway [29]. Consistent with SCAR's primary role in BG-2 morphogenesis, depletion of *SCAR* leads to the adoption of the shape 2, a shape that does not exist in a wild-type population (see electronic supplementary material, table S3).

In summary, we propose that Rho and Rac, and their downstream effectors, serve as a core pathway that establishes the basic template of morphologies explored by all eukaryotic cells. RhoGEFs and RhoGAPs act to couple morphogenesis to different environmental signals, whereas other cytoskeletal regulators act to fine-tune cell shape and elaborate upon the basic shapes established by Rho and Rac.

## 5. Material and methods

### 5.1. Software

Analyses were performed using Matlab.

### 5.2. Dataset

The dataset used in this study was generated in an image-based screen of 256 TCs, where genes were either systematically inhibited by RNAi or overexpressed by transient transfection. In a limited number of TCs, there is a combination of overexpression and RNAi [26]. Cells were stochastically labelled with EGFP to facilitate image segmentation. For all TCs, 145 features were measured for 12 061 cells.

### 5.3. Data transformation and reduction

All measured features are scaled between 0 and 1. After scaling, a constant of 0.01 is added to all measurements to make them non-zero and then a logarithmic transformation is applied. PCA is performed on the transformed data. The first three PCs analysed represent 59% of the variability in the original data and are considered for further analysis. Each other PC represented less than 5% of the variability in the original data and thus all have been discarded. Eigenvalues for this analysis are listed in the electronic supplementary material, tables S1 and S2.

### 5.4. Single-cell clustering

For both hierarchical clustering and Gaussian mixture models, clusters are computed by varying the number of clusters (*k*) between five and 30. Hierarchical clustering is performed with Euclidean distance and Ward linkage. Gaussian mixture modelling is performed 10 times for each *k* and the final clustering with the best log-likelihood value is chosen. The average of silhouette values is used to assess the quality of the computed models, and the model with the highest average silhouette score is chosen.

### 5.5. Clustering performance improvement

The chosen model has an average silhouette value of 0.7647. To improve the clustering performance, we divide the cells into two groups: cells with high silhouette values (more than 0.6) and cells with low silhouette values (less than 0.6). Next, we reclassify cells in the second group to those in the first group using KNN search, where *k* = 10. The final model achieves an average silhouette value of 0.7758.

### 5.6. Treatment condition heterogeneity profiles

For each TC, including the wild-type (cells expressing EGFP alone) population, the percentage of cells in each shape is computed, resulting in a vector describing the distribution of cells for each TC. These vectors are then normalized by subtracting the wild-type TCHP. All THCPs are listed in the electronic supplementary material, table S3.

### 5.7. Normalized treatment condition heterogeneity profile clustering

Normalized TCHPs are clustered by hierarchical clustering and complete linkage. The clustering that results in the highest degree of overlap with the clusters derived by Bakal *et al.* was chosen [26]. To measure the distance between two TCHPs, we weigh the difference between two TCHP vectors by the difference between the shapes’ average PC scores. The formula for the distance metric is
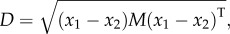
where *D* is the distance between two normalized TCHPs, *x*_1_ and *x*_2_ are normalized TCHP vectors, *M* is the square matrix of the Euclidean distances between the mean values of the first three PC scores for each shape, and the superscript T is the transposition operator.

### 5.8. Dependency analysis

We generate a dependency model of different shapes using Biolearn software (http://www.c2b2.columbia.edu/danapeerlab/html/biolearn.html). We scale the percentages of each shape across different TCs onto interval [0,1] for normalization. We then run Bayesian learning 500 times with normal gamma function. Across the 500 resulting models, we retain edges that are present in at least 60% of the models. We assign directionality to the edge based on the direction that appeared most frequently, even if the direction appeared in fewer models than edges with no direction.

### 5.9. Live-cell imaging

DM-BG2 cells (referred to as BG-2 cells in this paper) are cultured in Shields and Sang M3 insect media (Sigma), 10% fetal bovine serum (Serum), 10 μg ml^−1^ insulin (Sigma), 1% penicillin–streptomycin (Gibco) at room temperature. For experiments to validate the Bayesian inference model, brightfield images are acquired every 5 min for 180 min on a Nikon Ti microscope. Electronic supplementary material, movie S3 is a representative movie where transition frequency is analysed. For other experiments, cells are transfected with plasmids encoding *actin-GAL4* and *UAS-EGFP* using Effectene transfection reagent (Qiagen). In the electronic supplementary material, figures S1 and S3*a*, and movie S3, we use a plastic pipet tip to remove cells from a region of the plate and thus create a region of free space for BG-2 to migrate into.

## Supplementary Material

Supplemental Table 1

## Supplementary Material

Supplemental Table 2

## Supplementary Material

Supplemental Table 3
